# Supplementary material for: Interpretation of the Epigenetic Signature of Facioscapulohumeral Muscular Dystrophy in Light of Genotype-Phenotype Studies
Source: Int J Mol Sci. 2020 Apr 10;21(7):2635. doi: 10.3390/ijms21072635 (PMC7178248; doi:10.3390/ijms21072635)
Supplement: Supplementary file 1 [file Suppl. Figure 1.pdf › Suppl. Figure 1.pdf]

A

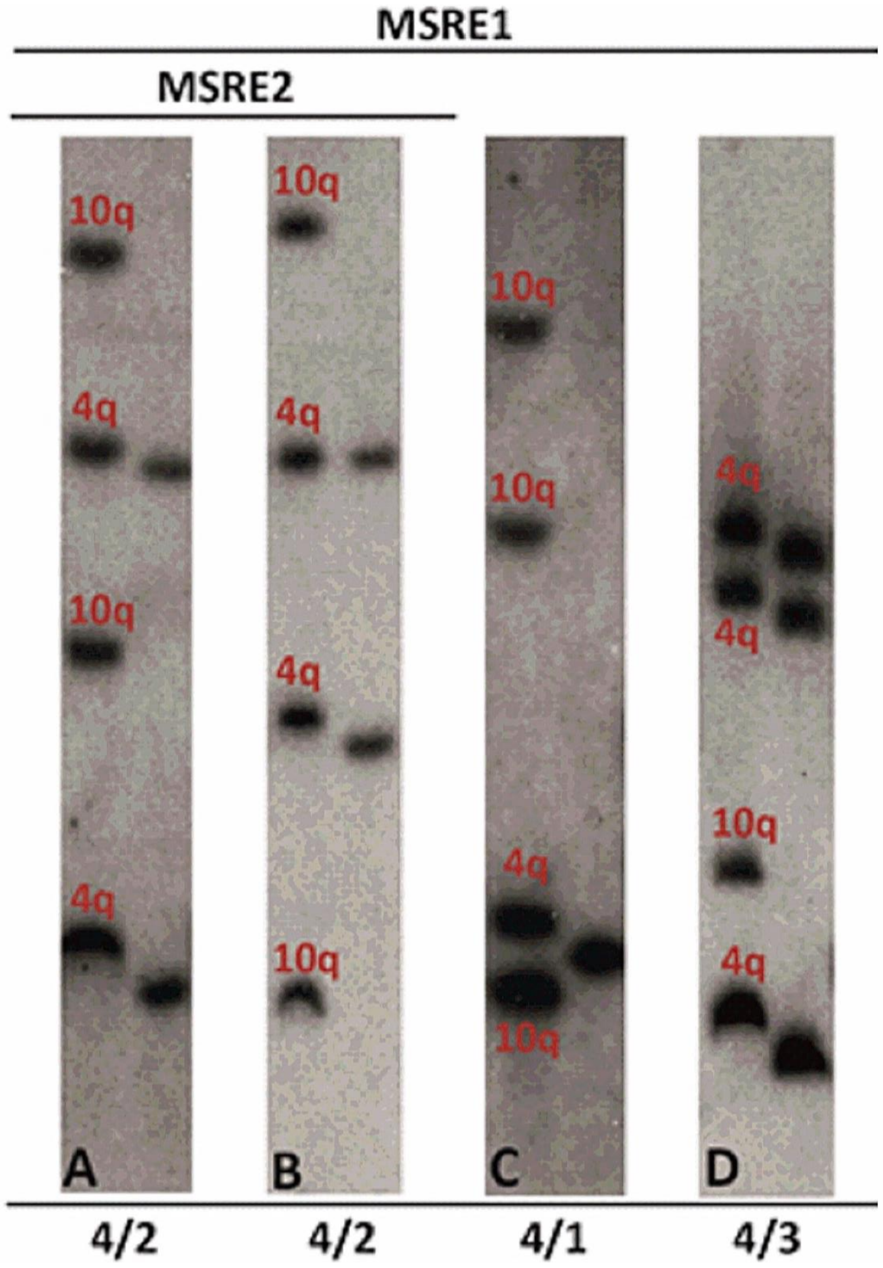

B

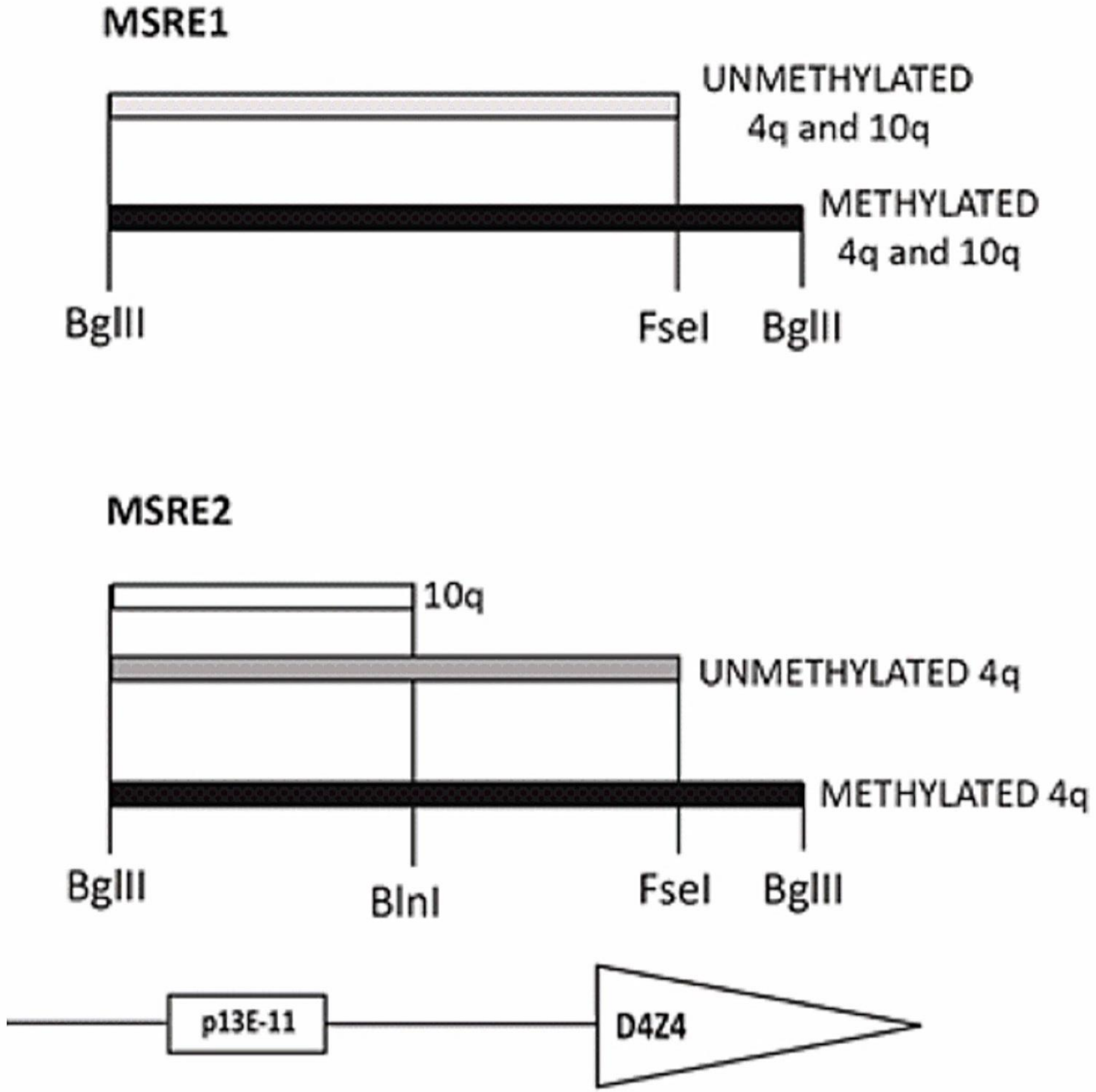

Suppl. Fig. 1
